# Optimization and performance testing of a sequence processing pipeline applied to detection of nonindigenous species

**DOI:** 10.1111/eva.12604

**Published:** 2018-02-20

**Authors:** Ryan Scott, Aibin Zhan, Emily A. Brown, Frédéric J. J. Chain, Melania E. Cristescu, Robin Gras, Hugh J. MacIsaac

**Affiliations:** ^1^ School of Computer Science University of Windsor Windsor ON Canada; ^2^ Research Centre for Eco‐Environmental Sciences Chinese Academy of Sciences Haidan District Beijing China; ^3^ Department of Biology McGill University Montreal QC Canada; ^4^ Great Lakes Institute for Environmental Research University of Windsor Windsor ON Canada; ^5^ Present address: Frédéric J. J. Chain, Department of Biological Sciences University of Massachusetts Lowell Lowell MA USA

**Keywords:** aquatic invasive species, biomonitoring, clustering, high‐throughput sequencing, metabarcoding, sequence processing

## Abstract

Genetic taxonomic assignment can be more sensitive than morphological taxonomic assignment, particularly for small, cryptic or rare species. Sequence processing is essential to taxonomic assignment, but can also produce errors because optimal parameters are not known a priori. Here, we explored how sequence processing parameters influence taxonomic assignment of 18S sequences from bulk zooplankton samples produced by 454 pyrosequencing. We optimized a sequence processing pipeline for two common research goals, estimation of species richness and early detection of aquatic invasive species (AIS), and then tested most optimal models’ performances through simulations. We tested 1,050 parameter sets on 18S sequences from 20 AIS to determine optimal parameters for each research goal. We tested optimized pipelines’ performances (detectability and sensitivity) by computationally inoculating sequences of 20 AIS into ten bulk zooplankton samples from ports across Canada. We found that optimal parameter selection generally depends on the research goal. However, regardless of research goal, we found that metazoan 18S sequences produced by 454 pyrosequencing should be trimmed to 375–400 bp and sequence quality filtering should be relaxed (1.5 ≤ maximum expected error ≤ 3.0, Phred score = 10). Clustering and denoising were only viable for estimating species richness, because these processing steps made some species undetectable at low sequence abundances which would not be useful for early detection of AIS. With parameter sets optimized for early detection of AIS, 90% of AIS were detected with fewer than 11 target sequences, regardless of whether clustering or denoising was used. Despite developments in next‐generation sequencing, sequence processing remains an important issue owing to difficulties in balancing false‐positive and false‐negative errors in metabarcoding data.

## 1 INTRODUCTION

Newly introduced populations that colonize novel ecosystems are usually small and inconspicuous (Leung, Drake, & Lodge, 2004). Detection of small and geographically restricted populations is technically challenging, yet critically important to management of aquatic invasive species (AIS; Beric & MacIsaac, 2015). Traditional early detection relies on techniques such as recruitment plates, video, scuba diving, trawling and netting—which may require tremendous amounts of sampling effort (Hoffman, Kelly, Trebitz, Peterson, & West, 2011)—typically followed by morphological identification. Furthermore, they may be ineffective if the introduced species is small, cryptic or morphologically variable (Ficetola, Miaud, Pompanon, & Taberlet, 2008). These attributes characterize many AIS, rendering monitoring of underwater environments an especially challenging task. Generally, genetic approaches are promising in the early detection of AIS, circumventing numerous challenges of traditional surveillance (Smart, Tingley, Weeks, van Rooyen, & McCarthy, 2015).

**Figure 1:**
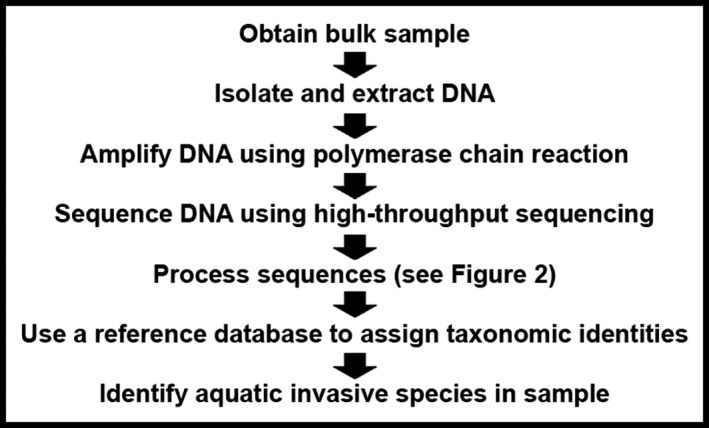
Flowchart of the general metazoan metabarcoding process applied to bulk sampling in the context of aquatic invasions. In this study, we focus on the computational aspects of the process (sequence processing, BLAST and identification of AIS)

When applied to complex communities, genetic detection of AIS or characterization of species composition typically involves sampling whole organisms (bulk sampling) or environmental DNA (eDNA) shed by them. In either case, a small “barcode” region of the genome (Hebert, Cywinska, Ball, & DeWaard, 2003) can be used to determine the taxonomic identity of mixed sequences (Cristescu, 2014). There are two genetic approaches to the detection of AIS. In the first, one must have a particular target (typically, a species) in mind (the “targeted” or “active” approach). Alternatively, metazoan metabarcoding (Fonseca et al., 2010) aims to recover a wide range of taxa in a community and passively discover AIS (the “passive” approach; Simmons, Tucker, Chadderton, Jerde, & Mahon, 2016). Metazoan metabarcoding typically involves the use of universal primers and PCR to amplify available genetic material aiming to recover all taxa from the captured sample. However, in reality, not all taxa are discovered with equal sensitivity due to primer design or choice, and consequently inconsistent amplification may occur (Creer et al., 2010; Xiong, Li, & Zhan, 2016). Owing to the complex process of metabarcoding metazoan bulk samples (Figure 1, applied to detection of AIS, described in Data S1), many potential sources of both false‐positive (type I) and false‐negative (type II) errors have been identified. A nonexhaustive list of potential sources of errors in this process includes primer design (Freeland, 2017), PCR (Piggott, 2016), next‐generation sequencing (Fonseca et al., 2010), sequence processing (Flynn, Brown, Chain, MacIsaac, & Cristescu, 2015), reference library preparation (Zhan, He et al., 2014) and taxonomic assignment inconsistencies, although it is difficult to quantify the impact of each (Xiong et al., 2016). Fortunately, by appropriately selecting parameters in computational sequence processing, the impact and frequency of errors can be reduced (Brown, Chain, Crease, MacIsaac, & Cristescu, 2015; Flynn et al., 2015; Zhan, Xiong, Song, & MacIsaac, 2014).

Over the last decade, several sequence processing suites have been developed, including USEARCH (Edgar, 2010), mothur (Schloss et al., 2009), QIIME (Caporaso et al., 2010) and RDP (Cole et al., 2014), each making simplifying assumptions that improve computational efficiency. Many of these suites share features, algorithms or even programs. Intraspecific genetic variation within barcode regions can exist, so many programs allow users to cluster sequences into operational taxonomic units (OTUs) based upon genetic similarity (Edgar, 2013; Schloss et al., 2009). OTUs are groups of sequences that share high similarity, typically at the species or genus level. UPARSE, which is built into the USEARCH program, can create clusters in order of decreasing sequence abundance after sequence dereplication (Edgar, 2013). Although the most abundant sequence may not represent the true center of a species, this approach is computationally efficient and is more effective than other approaches (such as UCLUST or hierarchical clustering of mothur; Edgar, 2013; Flynn et al., 2015). Other approaches to clustering—such as Bayesian (Hao, Jiang, & Chen, 2011), modularity‐based (Wang, Yao, Sun, & Mai, 2013) and agglomerative clustering (Mahé, Rognes, Quince, de Vargas, & Dunthorn, 2014)—may use different sequence identity definitions; that is, they penalize gaps in alignments differently. Several of these sequence processing suites have similar or shared features and algorithms; for example, the clustering algorithms in QIIME are strictly third‐party and some are closed source (Caporaso et al., 2010). USEARCH is comprehensive and allows sequence trimming, minimum Phred score (*Q*) filtering, maximum expected error (MEE) filtering, clustering, denoising (Edgar, 2016) and removal of sequences not meeting any arbitrary abundance threshold. These are all options that are regularly used in the related literature in some capacity, even in computational suites other than USEARCH (Bista et al., 2017; Bokulich et al., 2013; Brown, Chain, Zhan, MacIsaac, & Cristescu, 2016; Brown et al., 2015; Chain, Brown, MacIsaac, & Cristescu, 2016; Elbrecht & Leese, 2015; Flynn et al., 2015; Hänfling et al., 2016; Pawlowski, Esling, Lejzerowicz, Cedhagen, & Wilding, 2014; Port et al., 2016). USEARCH also has many other utilities for analysis after sequences have been processed, such as computation of diversity indices and phylogenetic analysis.

The objective of sequence processing is to improve the integrity of results, but it may also be a source of error if performed poorly (Brown et al., 2015; Flynn et al., 2015; Xiong et al., 2016). Parameter selection in sequence processing involves a delicate balance between false‐positive and false‐negative error (Zhan et al., 2013). With overly stringent quality filtration, for example, sequences that identify truly present taxa in a sample may be removed, leading one to incorrectly infer absence of these taxa (false‐negative error). On the other hand, insufficient filtration can lead to false‐positive errors, because in downstream analyses, erroneous sequences could map to species not present in the sample. Filtering is discussed here for illustrative purposes; all other components of the pipeline (clustering, denoising, length cutoffs, abundance thresholds, etc.) similarly participate in this balance between false positives and false negatives, and thus, parameter selection is not straightforward. The optimal parameter sets (which minimize either or both types of error) depend on the aim of the study and are usually not known prior to processing. Currently, users have limited knowledge on which to base parameter selection.

Although computational processing of sequences is an essential part of taxonomic assignment for genetic sequence data, very few studies have attempted to rigorously address the problem of parameter selection (i.e., Bokulich et al., 2013). Instead, few (or single) aspects of sequence processing have been previously tested—often with low resolution (i.e., Brown et al., 2015, 2016; Flynn et al., 2015; Pawlowski et al., 2014)—although numerous processing steps and parameter values interact to produce the resultant set of sequences or OTUs. Parameter selection also depends on the goals and methods of the study (identification of AIS, species richness estimation, eDNA, bulk sampling, etc.). Thus, there is a need to test a wide range of processing steps and parameter values in concert and for different research scenarios.

**Figure 2:**
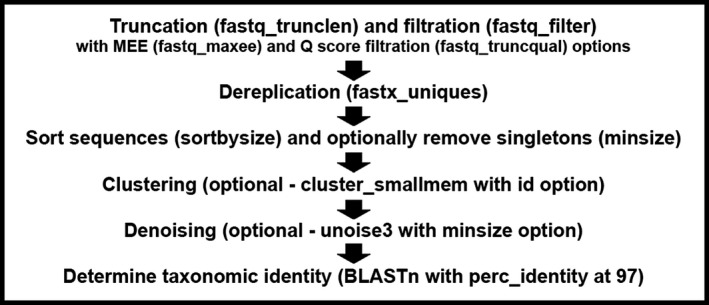
Flowchart of the sequence processing pipeline used in this study. Relevant USEARCH commands and options used are shown in parentheses. The first step combines sequence trimming (truncation) and quality filtration (Phred score—*Q*, and maximum expected error—MEE). In the next step, sequences are dereplicated. Next, the sequences are sorted in terms of decreasing abundance (necessary for clustering and denoising) and singletons are removed. Clustering or denoising of the sequences may subsequently be performed. Finally, BLASTn is used to perform taxonomic assignment with a minimum identity threshold of 97% using BLASTn defaults

We primarily sought to determine how users should select parameters when using a sequence processing pipeline (Figure 2) in a metazoan, bulk sample, metabarcoding context. Simultaneously, we wanted to determine whether and how research goals influence optimal parameter selection. Finally, we aimed to determine the performance of such a pipeline when parameters were appropriately selected given these research goals. Consequently, this study had two main investigations: *optimization*, in which we searched for optimal parameter selection for the computational sequence processing pipeline, and *performance testing*, in which we performed simulations to assess the performance of selected “most optimal parameter sets” in two ways, sensitivity and detectability (defined below under *Performance Testing*). In both parts of the study, we considered two common research applications of metabarcoding: accurate estimation of species richness and early detection of AIS. These research goals differ in how researchers will utilize sequence processing pipelines to shift the balance between protection against false positives and false negatives. Although it is always important to control for both types of errors, researchers estimating species richness via metabarcoding are typically concerned with minimizing both false positives and false negatives, while those involved in early detection of AIS are mainly concerned with minimizing false negatives.

## 2 MATERIALS AND METHODS

Below, we give a brief overview of our study. We then describe our sequence processing pipeline, introduce our sequence datasets, explain the optimization process and discuss our performance testing procedure.

First, we optimized a sequence processing and taxonomic assignment pipeline (Figure 2) employing USEARCH v10.0.240_i86linux32 (Edgar 2010) and BLASTn v2.6.0+ (Altschul et al. 1990) using a mock (i.e., deliberately assembled) community of sequences from 20 AIS obtained via 454 pyrosequencing. We used the USEARCH package because it is comprehensive, fully automatable through scripting, and exhibits strong performance and efficiency (Edgar, 2013). We optimized the pipeline separately for two common research goals: accurate estimation of species richness (which favors minimizing false negatives and false positives when sequences vary in abundance) and early detection of AIS (which favors sensitivity and minimizing false negatives, even for sequences of low abundance). This stage involved a search for parameter sets that generated OTUs that most accurately reflected the makeup of the mock community samples, which we described in detail below under the section “Optimization.” Secondly, we took some of the most optimal parameter sets from the optimization phase and tested their performance through simulation. We tested performance using 20 different AIS, community samples from 10 ports and the most effective 24 parameter sets (of 1,050 total parameter sets tested), allowing us to observe dependencies between these factors. This allowed us to make recommendations for sequence processing parameter selection from a more general standpoint.

### 2.1 Sequence processing

We defined a parameter set as a combination of sequence length, *Q* filter stringency, MEE filter stringency, clustering identity threshold (if clustering was used), denoising minimum sequence abundance (if denoising was used) and minimum sequence abundance after dereplication. The values we tested for each parameter can be found under *Optimization*. To elaborate, sequences shorter than the sequence length threshold were removed, while those longer than that length were trimmed accordingly. The *Q* filter we used was a minimum *Q* score filter, meaning that a sequence with any single base call with *Q* below the threshold was removed. The MEE filter computed the maximum number of expected errors across the entire sequence using *Q* scores of each base call. Sequences with an expected number of errors above the MEE threshold were removed. Clustering identity was the similarity threshold between an OTU's representative sequence and all other sequences in that OTU using UPARSE. Denoising in USEARCH (UNOISE3) considered sequence abundance and number of differences between sequences to predict whether a sequence was correct or not (Edgar, 2016). In UNOISE3, the probability of incorrectness of a sequence was computed based on the abundance skew ratio (ratio of abundance) and number of differences between it and other sequences already deemed correct, and sequences were compared in order of decreasing abundance for efficiency (see Edgar, 2016 for algorithm details). Denoising minimum abundance was the minimum abundance for a sequence to not be considered noise, which also affected abundance skew ratio ratios for retained sequences. With any given denoising minimum abundance, retained (but noisy) low‐abundance sequences counted toward abundances of their “correct” counterparts. This could impact classification of sequences at the denoising step and could also influence downstream abundance‐based analyses. Further, as the lower limit on sequence abundance was increased, remaining sequences could be classified as noisy or correct with greater confidence with the UNOISE3 algorithm (Edgar, 2016). We left the other clustering and denoising parameters to their default values. Minimum sequence abundance after dereplication was simplified by either allowing or removing singletons.

We used the same sequence processing procedure in both optimization and performance testing (Figure 2). We used USEARCH for all sequence processing. This procedure took as input a single FASTQ file, although it could also be adapted for merged paired reads. In the first step, we truncated sequences, removed those not meeting the length requirement and then filtered the sequences by quality. Next, we dereplicated and sorted sequences by abundance, which was necessary for the UPARSE clustering and UNOISE3 denoising algorithms built into USEARCH. In this step, if singletons were to be removed, only sequences with two or more replicates were retained. Whether clustering or denoising was performed or not was determined by the parameter set being tested (i.e., the iteration of the optimization stage or the selected parameter sets in performance testing). We did not test combining clustering and denoising due to computational constraints. A chimera detection algorithm is embedded in the denoising algorithm of USEARCH that we used (UNOISE3), so chimera detection occurred if denoising was performed using the defaults for UNOISE3. Once sequence processing was complete, we checked the resultant set of sequences (or OTU representative sequences) against precomputed BLAST results (see *Dataset preparation* below for BLAST precomputing procedure). All computing was performed on the Shared Hierarchical Academic Research Computing Network (SHARCNET).

### 2.2 Dataset preparation

We acquired four published metabarcoding datasets of 18S V4 rDNA sequences. The amplified fragment length was ≥400 bp for our target taxa. Primers for this marker effectively amplify a broad range of zooplankton taxa, making 18S a suitable marker for zooplankton metabarcoding studies (Zhan, Bailey, Heath, & MacIsaac, 2014). Conversely, the COI marker is highly variable for these taxa (sometimes, even in the primer binding sites) which may make it more suitable for studies taking the targeted approach than for metabarcoding highly divergent communities (Deagle, Jarman, Coissac, Pompanon, & Taberlet, 2014; Hatzenbuhler, Kelly, Martinson, Okum, & Pilgrim, 2017; Zhan, Bailey et al., 2014). The drawback of 18S is that due to lower variability, it may be more difficult to assign identity at the species level. For each dataset, we obtained unprocessed sequences in FASTQ format. The first dataset, which we called D1, was a mock community of 20 AIS obtained from bulk zooplankton samples, with derived sequences grouped by species. Preparation of this dataset is detailed (see Data S2).

In performance testing, we also utilized a dataset that consisted of V4 18S rDNA derived from bulk zooplankton samples from ten Canadian ports (Chain et al., 2016). We kept each of these samples separated by port and refer to this as D2 (Table S1). Sequences of D1 were computationally inoculated into samples from D2, as explained in more detail below under “Performance Testing.” Primers and tags were removed from all sequences. In cases where, after sequencing, the primer or tag of a sequence did not match any original primers or tags, the sequence was removed.

For optimization and performance testing, we needed to classify each sequence in D1 as correct, ambiguous or incorrect (see Data S3 for further details on sequence classification). A correct sequence was one that aligned best with a reference sequence of its true identity, with identity ≥97%, whether alignments to other taxa were tied in similarity score or not. An ambiguous sequence was one that aligned with a higher score to a reference sequence of a different taxon, although it still aligned to its correct taxon with identity ≥97%. An incorrect sequence aligned with a reference sequence of its true identity with identity <97%.

### 2.3 Optimization

We tested 1,050 parameter sets (see summary, Table S2). It is important to note that we tested 150 parameter sets without clustering or denoising, but tested 450 parameter sets with clustering and 450 with denoising because we explored three values for each clustering and denoising parameter. Testing fewer parameter sets without clustering or denoising implies that we explored a smaller space of possibilities for that method of processing, which can potentially lead to reduced observed optimality for this method. However, it was more important that, for each common parameter across the processing methods, we tested the same parameter values to keep the methods comparable. The parameters and values we tested were informed by related studies in the field and the characteristics of our sequence datasets (see Data S4 for parameters and values used in related studies).

**Figure 3:**
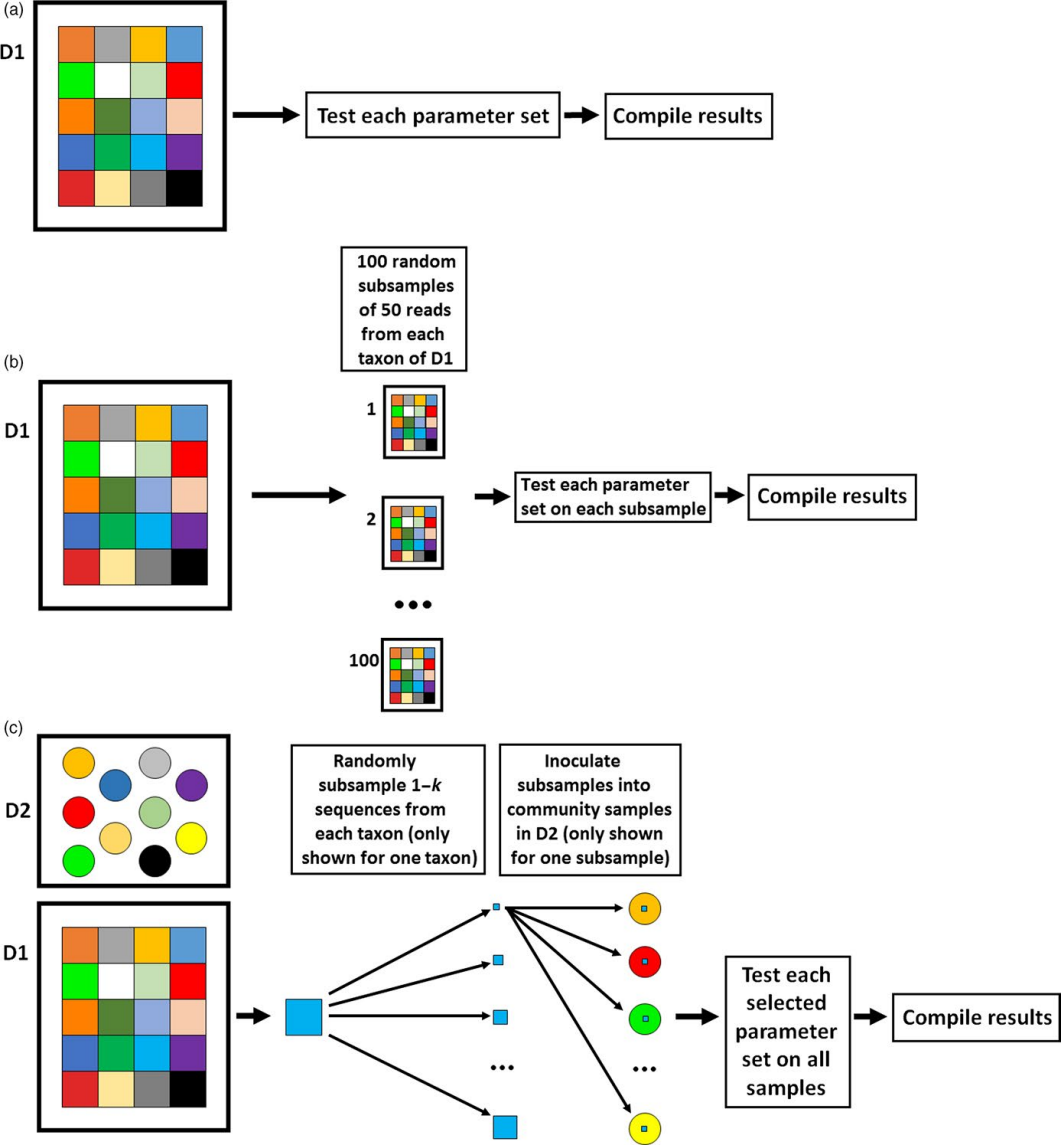
The optimization method for accurate species richness estimates (a), early detection of AIS (b) and the performance testing method (c). Different colored boxes represent different taxa in dataset 1 (D1), and different colored circles represent different community samples in dataset 2 (D2). For performance testing of parameter sets optimized for accurate species richness estimates, *k* = 100. For performance testing of parameter sets optimized for early detection of AIS,* k* = 50. For a given iteration *i*, where 1 ≤ *i* ≤ *k*, different random subsamples with *i* sequences from a given taxon were used to inoculate each community

Optimization consisted of two parts. In both parts, ranking of parameter sets was based on the number of correct, ambiguous, incorrect and redundant OTUs generated by the pipeline (see Data S5 for details of the ranking process). For each taxon, we classified only one OTU as correct or ambiguous and all other correct or ambiguous OTUs were reclassified as redundant (see Data S5 for specifics on redundant OTUs). Part I was designed to find parameter sets that most accurately estimated species richness (i.e., minimized false negatives and false positives with varied sequence abundances) from a bulk zooplankton sample (Figure 3a). Part II was designed to find parameter sets with high sensitivity (i.e., minimized false negatives with low sequence abundances, Figure 3b), which is more useful in the detection of AIS. In part I, we combined the samples from all 20 taxa from D1 to construct a single mock community sample. The number of sequences for each D1 taxon ranged from 200 to 46,915. In part II of optimization, we generated 100 samples, each consisting of 1,000 sequences. We generated these samples by randomly resampling D1, aggregating subsamples of 50 sequences from each taxon to form mock communities with low sequence abundance. Using only 50 sequences from each taxon forced the optimization process to favor more sensitive parameter sets—those that could successfully recover taxa even with low sequence abundance—which was more appropriate when minimization of false‐negative error was vital. In both part I and part II, we then tested all 1,050 parameter sets on all samples and computed the number of correct, ambiguous, incorrect and redundant OTUs generated by the pipeline using the given parameter set across all samples. Finally, we ranked the parameter sets according to the optimization ranking scheme (see Data S5 for details of the optimization ranking scheme).

To determine the concordance of parameter set rankings between the two research goals, we computed the Kendall rank correlation coefficient on the ranked parameter set lists for each sequence processing method. Furthermore, we determined the relative contribution to false‐negative and false‐positive errors of each of the parameters for six cases: three sequence processing methods across two research goals. In each case, we performed a multiple regression analysis using optimization results. The predictors were the parameter values, and the response variables were the number of correct + ambiguous OTUs (which indicates increasing false‐negative error as it decreases from 20) and the number of incorrect OTUs (which indicates increasing false‐positive error as it increases from zero). We standardized parameter values for each regression, which allowed us to use the magnitude of the regression coefficients to rank parameters by their relative contributions. In each case, we reported the regression coefficients (to indicate relative contribution) and associated *p* values (to indicate significance of their contributions).

### 2.4 Performance testing

We ran a series of simulations to test performance of the pipeline in detecting target sequences that were computationally inoculated into real bulk zooplankton samples using 24 selected parameter sets from optimization (Figure 3c). The “target” sequences were a subset of sequences all belonging to a single AIS from D1. We chose 12 parameter sets from optimization part I and 12 from part II. We did not simply choose the top 12 parameter sets from each part of optimization because many of the top parameter sets were quite similar. For both parts of the optimization stage, we chose four parameter sets for each processing method—clustering, denoising, and neither clustering nor denoising. We always chose the top parameter set for a processing method and subsequently selected parameter sets that performed the next best but were at least two parameters different from any other previously selected parameter set until we had a total of four parameter sets for that category. We conducted performance testing in two parts, mirroring the two parts of optimization. In part I, a simulation consisted of inoculating each port sample in D2 with target sequences, iterating from 1 to 100 randomly selected sequences of a target taxon from D1. We did this for every taxon in D1. We then ran the pipeline with all selected parameter sets from optimization part I on the simulated data. For each combination of target taxon, port and parameter set, we performed 25 simulations. For each simulation, we recorded if the target was detected with up to 100 sequences inoculated into the sample and, if so, how many sequences were required to detect it. Therefore, we defined two measures of performance: detectability and sensitivity. Detectability was defined as the ratio of simulations in which the target was found given some number of target sequences inoculated into a community sample. Sensitivity was defined as the number of sequences required to detect the target. Sensitivity was not recorded if the target was not detected. Part II was identical to part one, except we used selected parameter sets from optimization part II and inoculated only up to 50 sequences of the target into the sample because the parameter sets from optimization part II were expected to be far more sensitive. We inoculated up to 50 sequences of the target due to computational constraints and because we found in preliminary work that if the target was not found with 50 sequences in the sample, it was likely undetectable.

## 3 RESULTS

### 3.1 Optimization

Classification of sequences prior to optimization revealed that D1 could yield, at most, 1,484 incorrect OTUs and trimming alone could be responsible for false‐negative error (see Data S6 for classification of sequences and OTUs during optimization). The most optimal parameter sets favored longer sequences with relatively weak filtering. For example, of the top 20 parameter sets from each category (clustering, denoising, or neither, for estimation of species richness or early detection of AIS—120 parameter sets in total), 106/120 (88.3%) trimmed sequences at length ≥375 bp. Trimming at shorter lengths was only viable if no clustering or denoising was performed, and even then it was suboptimal. No top 20 parameter sets in any category used a *Q* filter with strength >10. Of top ten parameter sets from each category, the mean MEE filter was 2.23, which was relaxed with respect to the range tested and relative to the literature (Bista et al., 2017; Brown et al., 2015; Flynn et al., 2015; Port et al., 2016). When aiming to optimize species richness estimation, the MEE filter had a mean of 2.12 (Table S3, selected parameter sets—see supporting information for full optimization results), whereas for early detection of AIS, it was 2.33 (Table S4, selected parameter sets). When denoising, the MEE filter in top ten parameter sets was more relaxed, particularly for early detection of AIS (mean MEE = 2.60). The top 12 parameter sets for accurate species richness estimation for pipelines without clustering or denoising all discarded singletons, as did the top five optimized for early detection of AIS. For pipelines involving clustering, the top eight parameter sets discarded singletons when seeking to optimize species richness estimation. Conversely, the top nine parameter sets with clustering kept singletons when optimizing for early detection of AIS. For denoising, keeping or discarding singletons did not matter because the minimum denoising abundance threshold tested was two. Using clustering, the top 18 parameter sets for accurate species richness estimation used an identity threshold of 99%, whereas the top 24 parameter sets for early detection of AIS also used an identity of 99%. For denoising, the top 14 parameter sets for species richness estimation used a minimum abundance threshold of eight, whereas the top 12 parameter sets for early detection of AIS used a threshold of two sequences.

We observed concordance of parameter set rankings determined by optimization for the two research goals. When clustering was used, the Kendall tau was 0.80, signifying strong concordance (*p *< .001). The Kendall tau was 0.79 when denoising was used and 0.77 when no clustering nor denoising was used (*p *< .001 in each case). Multiple regression analysis determined that parameter selection accounted for less variation in the number of correct + ambiguous OTUs recovered when determining species richness (80%, 89% and 80%, when clustering, denoising or neither, respectively; see Table S5 for summary of multiple regression results) than when aiming for early detection of AIS (95%, 94% and 95%, respectively). Conversely, given either research goal, parameter selection accounted for comparable amounts of variation in the number of incorrect OTUs recovered (41%, 51% and 47% for estimation of species richness, 48%, 55% and 50% for early detection of AIS).

We found that, regardless of research goal or processing method, *Q* filter strength most strongly determined both the number of correct + ambiguous OTUs recovered and the number of incorrect OTUs recovered (*p *< .001 in each case; ranking of parameter importance, Table 1; coefficient and *p* values, Table S5). Generally, MEE filtration had little contribution to correct + ambiguous OTU counts and was most significant (*p *= .13) when denoising was used for early detection of AIS. Conversely, MEE filtration was generally important in reducing the number of incorrect OTUs (*p *< .05 in all cases, except when denoising for species richness estimates), always ranking third except when denoising was performed (in which case it ranked fourth). Sequence length was generally important in determining correct + ambiguous OTUs (*p *< .05 except when no clustering nor denoising was used for species richness estimation), with a mean rank of three. On the other hand, sequence length generally had a weaker contribution to the number of incorrect OTUs (mean rank = 3.8) and was insignificant when neither clustering nor denoising was used for either research goal (*p* > .05 in both cases). Keeping or discarding singletons was insignificant in determining either OTU count (correct + ambiguous or incorrect) when denoising was used, for either research goal. Otherwise, its mean rank was 2.5 for recovering correct + ambiguous OTUs and 2.0 in all cases for recovering incorrect OTUs (*p *< .05 in all cases except when no clustering or denoising was used for estimation of species richness). When clustering was used, identity threshold ranked fourth for each research goal and OTU count, and was not significant in determining the number of correct + ambiguous OTUs (otherwise, *p *< .05). Conversely, clustering identity threshold strongly impacted the number of incorrect OTUs (*p *< .05 for each research goal). When denoising was used, the denoising minimum abundance had a significant impact in all cases (*p *< .05) with a mean rank of 2.3.

**Table 1:** Parameter rankings (denoted as “Rank”) for each goal (estimation of species richness or early detection of AIS) and for each sequence processing method (clustering, denoising or neither), in terms of relative impact on the two optimization criteria (correct + ambiguous OTUs, incorrect OTUs). “*Q*” denotes *Q* filter, “Length” denotes sequence length cutoff, “Singletons” denotes whether singletons were kept or discarded, “MEE” denotes maximum expected error filter, “ID” denotes clustering identity threshold, and “DMA” denotes denoising minimum abundance. See Table S5 for coefficients and *p* values related to parameter impacts, determined by standardized multiple regression. Asterisk denotes significant impact at α = .05

Rank	Correct + Ambiguous	Incorrect	Rank	Correct + Ambiguous	Incorrect
Species richness			Early detection of AIS		
Clustering			Clustering		
1	Q*	Q*	1	Q*	Q*
2	Length*	Singletons*	2	Singletons*	Singletons*
3	Singletons*	MEE*	3	Length*	MEE*
4	ID	ID*	4	ID	ID*
5	MEE	Length*	5	MEE	Length*
Denoising			Denoising		
1	Q*	Q*	1	Q*	Q*
2	DMA*	DMA*	2	DMA*	Length*
3	Length*	Length*	3	Length*	DMA*
4	MEE	MEE	4	MEE	MEE*
5	Singletons	Singletons	5	Singletons	Singletons
Neither			Neither		
1	Q*	Q*	1	Q*	Q*
2	Length	Singletons*	2	Singletons*	Singletons*
3	Singletons	MEE*	3	Length*	MEE*
4	MEE	Length	4	MEE	Length

### 3.2 Performance testing

Distributions of the number of sequences necessary to detect targets varied by parameter set and exhibited positive skewness (i.e., parameter sets optimized for early detection of AIS without clustering or denoising, Figure S1—long tails above the mean and fewer samples below). No distribution for any single taxon, port or parameter set (optimized for either research goal) was normal (Kolmogorov–Smirnov test for normality, *p *< .05 in all cases), yielding generally high variance.

**Figure 4:**
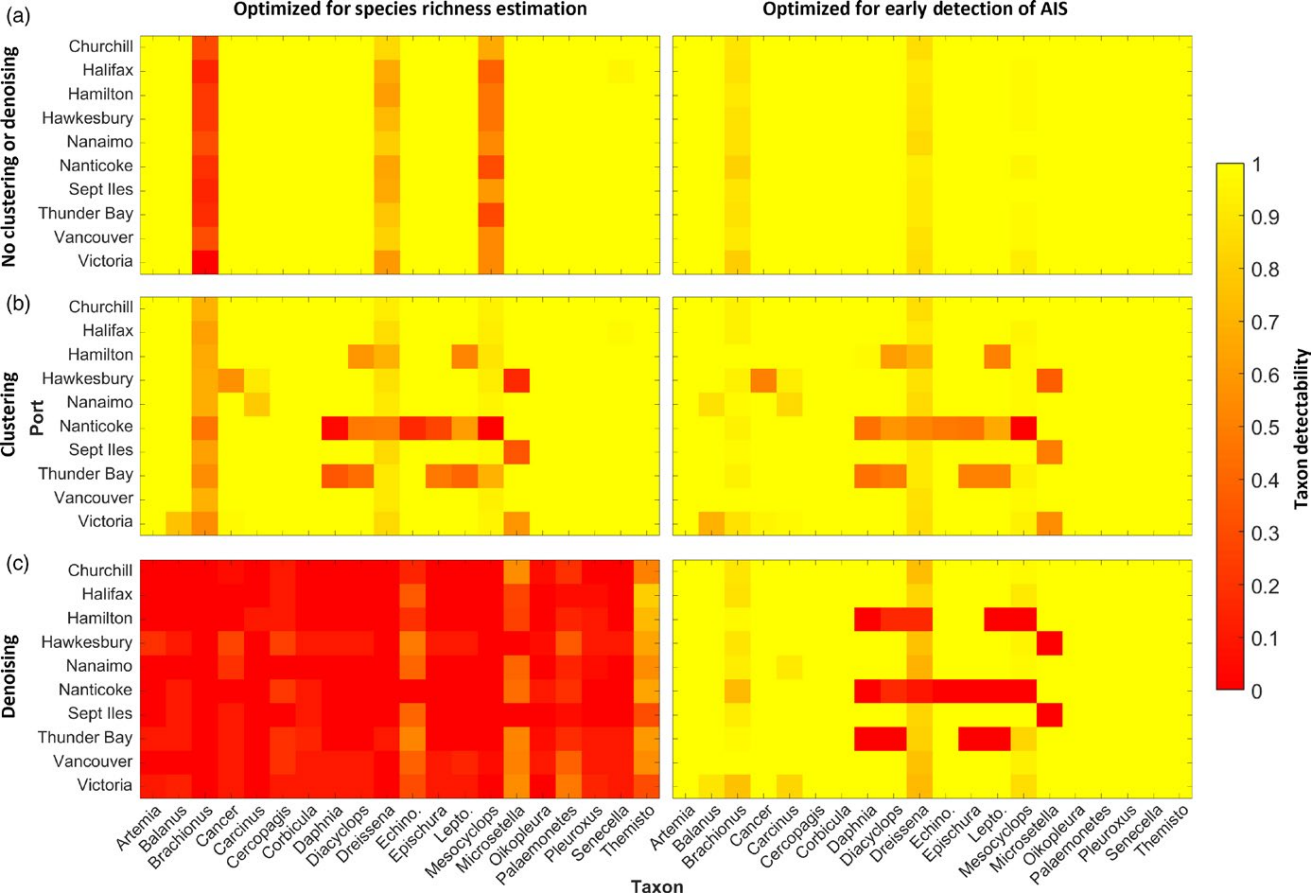
Detectability of taxa in mock samples, given as a value between 0 (no detectability of the target taxon at the port; red) and 1 (perfect detectability of the target taxon at the port; yellow) for parameter sets optimized for estimation of species richness (left column) and parameter sets optimized for early detection of AIS (right column) using no clustering or denoising (a), clustering (b) and denoising (c), across all ports and taxa, with 10 sequences of each taxon inoculated into the original port sample. Detectability for a given port and taxon was computed using all replicates involving the given port and taxon (i.e., across all parameter sets tested). See Table S6 for species names

For parameter sets optimized for species richness estimation, detectability with 10 target sequences inoculated into the port sample was nearly perfect without clustering or denoising for all taxa aside from *Brachionus*,* Dreissena* and *Mesocyclops* (Figure 4a, left column). The latter species detectability was poor owing to the low quality of their sequences relative to those for other taxa. A similar pattern was observed with clustering, although several ports (e.g., Hamilton, Nanticoke and Thunder Bay) yielded low detectability for several taxa (Figure 4b, left column). Detectability across all combinations of port and taxon was very poor when denoising was used (Figure 4c, left column).

A similar but slightly improved detectability pattern was observed for parameter sets optimized for early detection of AIS not using clustering or denoising, when compared to those optimized for species richness (Figure 4a). Detectability of *Brachionus* and *Mesocyclops* was significantly improved across ports for parameter sets using clustering optimized for early detection of AIS when compared to those optimized for estimation of species richness (*p *< .001); otherwise, there were no significant differences in detection for any port or taxon (*p* > .05). A similar detectability pattern was observed for clustering using parameter sets optimized for early detection of AIS as compared to those optimized for estimation of species richness (Figure 4b), although a slight overall improvement was observed (only *Brachionus* detectability was significantly improved; *p *< .001). Overall, we observed high variation in recovery ratio across ports and target when clustering or denoising was performed with parameter sets optimized for early detection of AIS (Figure 4b,c, right column). For example, the freshwater ports of Nanticoke, Thunder Bay and Hamilton yielded low detectability, as recovery ratios were only 0.806, 0.887 and 0.939, respectively, when clustering was used. When denoising, the respective recovery ratios were even lower, only 0.648, 0.782 and 0.765. We observed no cases where a taxon could not be detected if 10 target sequences were present in the sample when clustering was optimized for early detection of AIS. Although the pattern for denoising was similar to that of clustering, many combinations of taxon and port yielded no detectability (Figure 4c, right column). Nevertheless, denoising parameter sets optimized for early detection of AIS yielded a significant improvement in detectability over those optimized for estimation of species richness for all taxa and all ports (*p *< .05).

**Figure 5:**
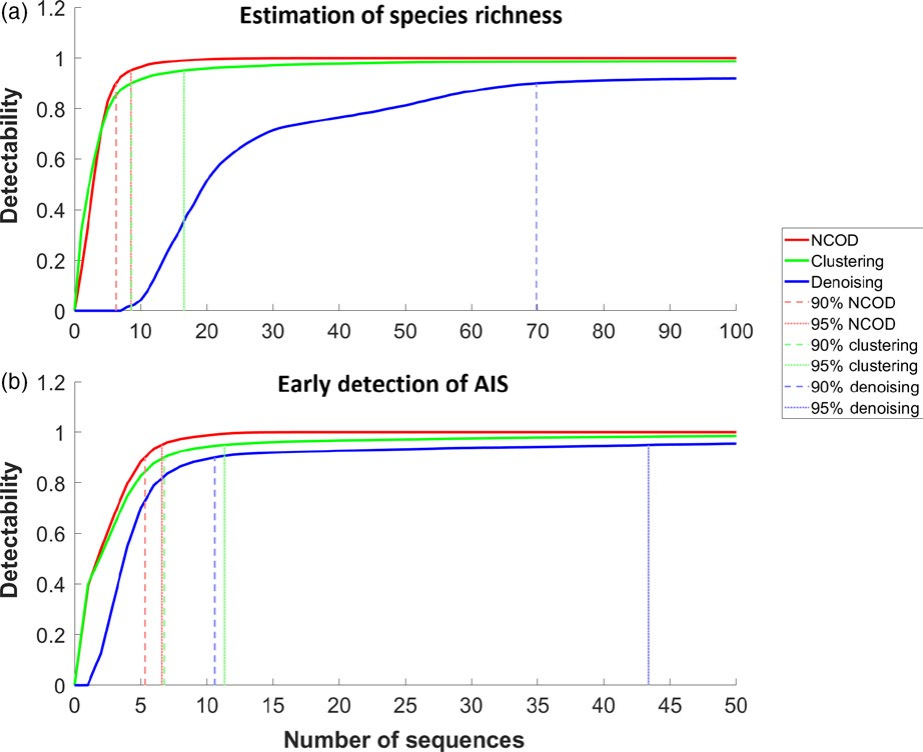
Overall detection probability of taxa for parameter sets optimized for estimation of species richness (a) and early detection of AIS (b) using no clustering or denoising (“NCOD”—red), clustering (green) and denoising (blue), per number of target sequences inserted into the original sample. Detection probability was computed using all combinations of taxon, port and parameter sets, across 25 replicates. Shown as dotted lines are 90% and 95% detection for each sequence processing method. Note the difference in *x*‐axis labels. For estimation of species richness using denoising, 95% detection was not achieved

Using parameter sets optimized for species richness estimation, detectability confidence reached 90% and 95% with the fewest sequences required using pipelines without clustering or denoising (Figure 5a). For example, on average 6.3 and 8.5 sequences were required to detect the target in 90% and 95% of replicates, respectively, when neither clustering nor denoising was used. With clustering, these values rose to 8.6 and 16.6 sequences, respectively. Denoising performed much worse, requiring 69.8 target sequences to reach 90% detectability while 95% detectability was unattainable. Detectability confidence was maximized in parameter sets optimized for early detection of AIS when clustering and denoising were not performed (Figure 5b). Without clustering or denoising, only 5.3 and 6.6 sequences were required for 90% and 95% detectability, respectively, 15.2% and 22.6% lower than when parameter sets were optimized for species richness estimates. These values rose to 6.8 and 11.3 target sequences, respectively, when clustering was used (11.2% and 31.8% lower than parameter sets optimized for species richness estimates, respectively), and 10.6 and 43.4 target sequences when denoising was used (84.9% fewer sequences for the 90% interval than parameter sets optimized for species richness estimates).

With parameter sets optimized for species richness estimation, sensitivity was far worse if denoising was used than if clustering or neither clustering nor denoising was used. Without clustering or denoising, only 3.9 (*SD* = 3.1) sequences were required to detect the target. This increased to 4.5 (*SD* = 7.0) sequences when clustering, and to 25.3 (*SD* = 16.4) when denoising. As expected, sensitivity improved with the top parameter sets that had been optimized for early detection of AIS. We found that 3.6 (*SD* = 4.9) reads were required to detect AIS (when they were detectable) using clustering, whereas denoising required 5.5 (*SD* = 5.8) reads. Without clustering or denoising, the pipeline was very sensitive, requiring only 2.9 (*SD* = 2.2) sequences. With clustering, we detected the AIS in only 98.5% of cases with 50 sequences inoculated. In contrast, denoising and neither clustering nor denoising detected the AIS in 95.5% and 100% of cases, respectively.

**Figure 6:**
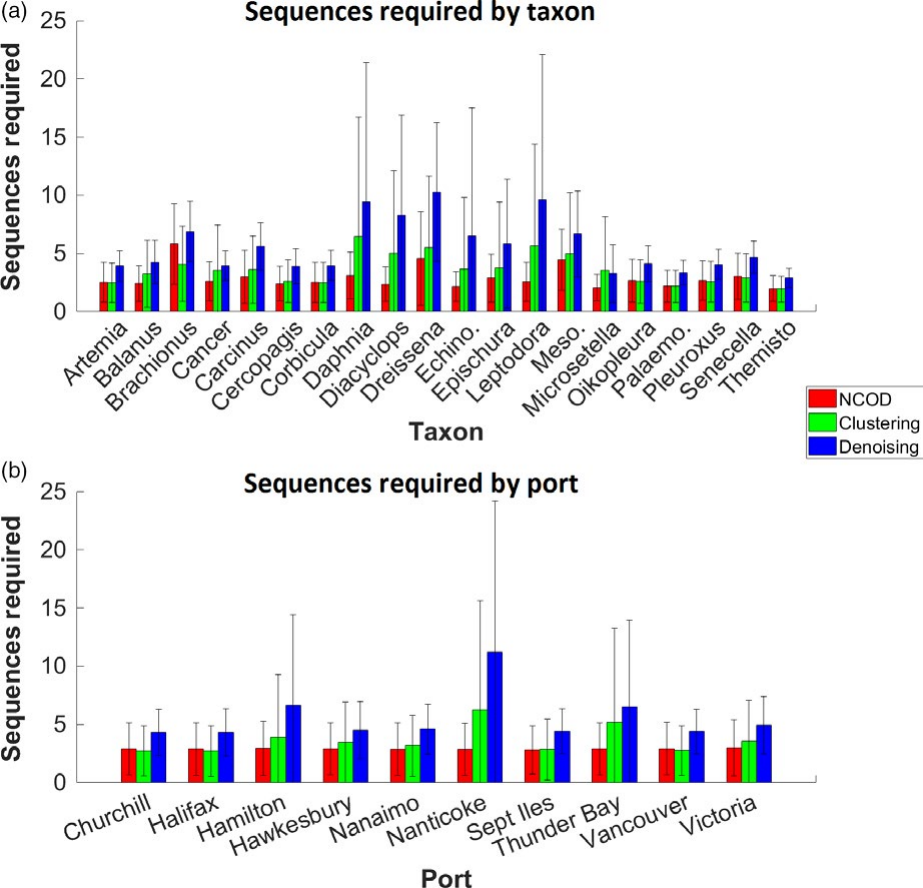
The sensitivity per taxon across all ports (a) and per port across all taxa (b), for parameter sets optimized for early detection of AIS using no clustering or denoising (“NCOD”—red), clustering (green) and denoising (blue). Error bars show standard deviation from the mean. See Table S6 for species names

For parameter sets optimized for early detection of AIS, four taxa (*Daphnia*,* Diacyclops*,* Dreissena* and *Leptodiaptomus*; Figure 6a—sensitivity for parameter sets optimized for early detection of AIS across taxa) required more than five sequences to be detected if clustering was used. This value rose to nine taxa if denoising was used, with the highly invasive *Dreissena* requiring the most sequences (mean 10.3; *SD* = 6.0). Without either clustering or denoising, only one taxon (*Brachionus*) required more than five sequences for detection (5.8; *SD* = 3.5). Variance in sensitivity was greater in taxa that yielded reduced sensitivity.

Using parameter sets optimized for early detection of AIS, we found that sensitivity varied little across ports (Figure 6b; sensitivity for parameter sets optimized for early detection of AIS across ports) except for Nanticoke when clustering (sequences required = 6.3; *SD* = 9.3) or denoising (sequences required = 11.2; *SD* = 12.9) was performed. Hamilton and Thunder Bay also yielded relatively lower sensitivity when clustering was performed, requiring 3.9 (*SD* = 5.4) and 5.2 (*SD* = 8.1) sequences, respectively, or 6.6 (*SD* = 7.8) and 6.5 (*SD* = 7.5) sequences when denoising was performed. Sensitivity across ports was very consistent with or without clustering and denoising.

## 4 DISCUSSION

In this study, we sought to assist users to optimally select processing steps and parameter values for sequence processing pipelines during metabarcoding of bulk zooplankton samples for the 18S marker on the 454 platform. Generally, we observed that trimming sequences to 375–400 bp was most favorable when a 400‐ to 600‐bp fragment was sequenced, and mild sequence quality filtration (1.5 ≤ MEE ≤ 3.0, *Q* = 10) worked best when overall sequence quality varied across samples (see summary of our findings on optimal parameter selection in Table 2). In optimization, denoising outperformed pipelines using clustering or neither clustering nor denoising regardless of the research objective. However, performance testing revealed that sequences—particularly at low abundance—of some taxa could wrongly be classified as noise during denoising, which resulted in false‐negative errors (see Figure 4c). Denoising pipelines also yielded very different distributions for sensitivity when compared to those that used clustering or neither clustering nor denoising. Denoising could drastically reduce sensitivity, particularly if the minimum abundance threshold for denoising was high (eight sequences). However, a high denoising minimum abundance threshold did reduce false‐positive errors, which indicated that it was useful for species richness estimates but not for early detection of AIS (when sensitivity and detectability are imperative). Naturally, without clustering or denoising, the pipeline was most sensitive and yielded highest detectability, as the AIS targets were detected in every case. Both clustering and denoising reduced false‐positive errors in optimization; however, these errors could be mitigated with further processing, so skipping clustering and denoising proved the best way to process metabarcoding sequences for the early detection of AIS.

**Table 2:** Summary of optimal sequence processing pipeline parameter selection for zooplankton 18S metabarcoding, given two research goals: estimation of species richness and early detection of AIS. “*Q*” denotes *Q* filter, “Length” denotes sequence length cutoff, “Singletons” denotes whether singletons should be kept or discarded, “MEE” denotes maximum expected error filter, and NCOD denotes “No Clustering or Denoising”. Note that keeping or discarding singletons in the early detection of AIS depends on whether the user will be clustering the data or not

Parameter/Option	Estimation of species richness	Early detection of AIS
Length	375–400 bp	375–400 bp
*Q*	10	10
MEE	1.5–2.5	2–3
Singletons	Discard	Depends on processing method. NCOD? Discard. Clustering? Keep
Clustering identity	99%	99%
Denoising minimum abundance	8	2
Processing method	Denoising	No clustering or denoising

Our study is the first to optimize such a sequence processing pipeline for metazoan bulk sample metabarcoding. In addition, we tested 1,050 parameter combinations for two different research objectives (i.e., estimating species richness and early detection of AIS). Other studies have focused on a single aspect of the sequence processing pipeline (Brown et al., 2015; Zhan, Xiong et al., 2014), tested relatively few combinations of parameters (Flynn et al., 2015), tested the ordering of processing steps (May, Abeln, Crielaard, Heringa, & Brandt, 2014) or tested bulk sample processing prior to sequencing, with mostly fixed sequence processing parameters (Piggott, 2016; Zhan et al., 2013). Brown et al. (2015) focused specifically on clustering sequence identity and found that a 97% identity threshold was sufficient in UPARSE to recover most taxa. Testing many parameter combinations also allowed us to explore interdependency between parameters and processing methods, even though it was computationally intensive. For example, even with high parallelization (~200 concurrent runs) of optimization and performance testing, the computational time required for this project was approximately 2 months on a high‐performance computing network (with CPU speeds of 2.2–2.7 GHz).

Further, our study is novel in that we tested the performance of optimized pipelines by computationally inoculating sequences of 20 species into real community samples to determine what can be expected for sensitivity and detectability given different combinations of community structure and ecosystem. In related work, Zhan, Xiong et al. (2014) spiked biomass of two AIS into two community samples. They found that relationships between false‐negative errors and exclusion of singletons, doubletons and tripletons with varied Phred score filters and biomass of target species spiked into real community samples. With strong filtering (*Q* = 30), spiked biomass of the marine scallop *Argopecten irradians* could not be detected in a real freshwater sample collected at Nanticoke, Lake Erie, although doubletons were usually recovered provided relatively weak filtering was carried out (*Q* ≤ 20) and sufficient biomass was present (Zhan, Xiong et al., 2014). Flynn et al. (2015) tested the ability of a similar pipeline to determine species richness of a mock zooplankton community using relaxed (length 250–600 bp, average *Q* ≤ 20) and stringent (length ≥ 400 bp, MEE ≤ 0.5) filtering methods, in combination with three different clustering algorithms with fixed clustering identity (97%). They concluded that UPARSE creates clusters most precisely and that stringent filtering was needed to accurately describe species richness. With a deeper optimization of this pipeline, we have corroborated their suggestions with respect to sequence length; however, our findings indicate that filtration can be more relaxed than they suggested. They also speculated that relaxed filtering might be necessary to recover rare taxa or sequences (i.e., in detection of AIS), a finding we explicitly tested and confirmed in this study.

Here, optimization of the pipeline revealed that keeping singletons generally did not reduce false‐negative errors except when using clustering in the context of early detection of AIS (in which the best nine parameter sets all kept singletons). Otherwise, removing singletons was a simple and uncostly means of reducing false‐positive errors. Generally, during optimization, retaining singletons increased redundancy and false‐positive errors without decreasing corresponding false‐negative errors. Although singletons could represent extremely rare taxa (see Brown et al., 2015; Zhan et al., 2013), they were more likely to be artifacts (Edgar, 2013; Flynn et al., 2015). Owing to the high sensitivity of the pipeline despite removal of singletons, we recommend that the advantages of reduced redundancy and false‐positive errors outweigh the disadvantage of slightly reduced sensitivity. Thus, singletons can generally be removed with little negative impact.

Previous studies covering different taxa, amplified fragments and applications have utilized sequence processing strategies that included more stringent *Q* filtering, typically between 20 and 30 (Bista et al., 2017; Elbrecht & Leese, 2015; Hänfling et al., 2016). In our study, with moderate filtering (*Q* ≥ 20, MEE ≤ 1.5), and especially at longer sequence lengths, all sequences of some species (particularly *Brachionus* and *Mesocyclops*) were removed, resulting in false‐negative errors whether the aim was to estimate species richness or to maximize sensitivity. This finding corroborated that of Zhan, He et al. (2014), who noted that rare taxa were more likely to be lost with increasing *Q* filter strength and informational sequences (those that represented otherwise undetected taxa) were removed at any stringency. Relaxed filtration allowed longer sequences to be analyzed downstream, as sequence quality generally decreased with sequence length. This is important because longer sequences generally provided greater taxonomic resolution and accuracy, allowing more appropriate definition of clusters (if clustering is used), more appropriate classification of a read as noisy (if denoising is used), and more accurate taxonomic assignment during BLAST. The downside of relaxed filtration was that it can increase false‐positive error.

We found that the most optimal parameter sets for estimating species richness allowed slightly more stringent filtration, which corroborated findings of Flynn et al. (2015). If the aim of the study is to accurately estimate species richness, sacrificing sensitivity and detectability (i.e., increasing false‐negative error) to decrease false‐positive error is justifiable. However, users should not increase the stringency of the *Q* filter as it is extremely sensitive and will remove a sequence if it has a single low‐quality base call. Conversely, if the objective is the early detection of AIS, false‐negative error is typically more costly than false‐positive error (a false‐positive error can potentially be mitigated downstream, e.g., when identifying sequences in BLAST), so filtration should be relaxed. Therefore, with respect to filtration and sequence length, we recommend mild MEE filtration (1.5–2.5 for species richness estimation, 2.0–3.0 for early detection of AIS), relaxed *Q* filtration (10 at most) and trimming sequences ≥375 bp. The upper bound on MEE and lower bound on *Q* filtration holds regardless of sequencing platform, as we used 454 pyrosequencing but cutting‐edge sequencers may improve read quality. The lower bound on MEE filtration could be reduced with newer sequencing technology, but *Q* filtration strength should not be increased for the reasons outlined above. The optimal sequence length depends on the amplified fragment and the length of sequences in the sample (which depends on sampling method and sequencing technology). Our amplified fragment was at least ~400 bp in target taxa—and 98% of target sequences were ≥400 bp—because we used 454 pyrosequencing of DNA extracted from bulk samples (eDNA sequences will likely be shorter due to degradation). Hence, it is sensible that our optimal sequence length (375–400 bp) was close to the minimum amplified fragment length in our taxa; taxonomic resolution was maximized while very few sequences were wrongfully excluded due to failing to meet the length cutoff. In studies where most sequences reach the minimum amplified fragment length in target taxa, we recommend using a length cutoff of approximately 90%–100% of the minimum amplified fragment length in target taxa.

We found that both clustering and denoising were useful in reducing false positives in the estimation of species richness. However, both should be avoided in the context of early detection of AIS because both sensitivity and detectability were reduced. We also found that a 99% clustering identity threshold was more optimal than the commonly used 97% identity threshold for bulk zooplankton 18S metabarcoding for either research goal, and a denoising minimum abundance threshold of 8 was best for estimation species richness (see Data S7 for a more detailed discussion of clustering and denoising).

Application of next‐generation sequencing in surveillance of AIS requires careful consideration of many options including sequencing technology, genetic marker and computational pipeline. Choice in sequencing technology has complex implications, manifested primarily in differences in sequence quality and length. We used 454 pyrosequencing in our study, although newer sequencing platforms could reduce sequencing errors. When this pipeline is used to determine species richness, one can potentially utilize more stringent filtering, although two or three base call errors in a sequence of length ≥375 bp are unlikely to cause a serious problem. Regardless, longer sequences improve taxonomic resolution and weaker filtration allows rare (and potentially otherwise undetectable) taxa to be discovered; thus, care must be taken to not filter too strongly in the context of surveillance for AIS.

With respect to marker choice, we used 18S in our study but COI has shown higher sequence variability and improved taxonomic assignment (Hatzenbuhler et al., 2017; Tang et al., 2012; Zhan, Bailey et al., 2014). This variability can be a double‐edged sword; as it is apparent even in primer binding sites, COI can have issues with primer generality (Deagle et al., 2014; Ficetola et al., 2010; Hatzenbuhler et al., 2017; Zhan, Bailey et al., 2014). Consequently, false‐negative errors may be more likely to occur because of inconsistent amplification which would be particularly detrimental to early detection of AIS. In the metabarcoding context, the variability of COI relative to 18S may impact sequence clustering, denoising and taxonomic assignment (e.g., through BLAST). With a higher‐resolution marker, sequences of different species will be more likely to be split into different OTUs during clustering (given some arbitrary identity threshold) and some sequences when denoising may be less likely to be considered noise because of increased sequence divergence. Downstream, taxonomic assignment in BLAST may be more confident for some taxa when using COI. Therefore, higher‐resolution markers could increase sensitivity and reduce false negatives whether clustering or denoising is used (because of the aforementioned advantages in sequence processing). However, even with a higher‐resolution marker (for example COI), we do not recommend clustering or denoising when conducting early detection of AIS for the reasons mentioned above. Many computational sequence processing suites offer similar (if not identical) features or algorithms for trimming, filtering, clustering and denoising (Caporaso et al., 2010; Cole et al., 2014; Edgar, 2010; Schloss et al., 2009). Consequently, many of our findings are generalizable to different sequence processing suites.

Regardless of marker and despite advancements in next‐generation sequencing technologies, sequence quality and processing are, and will continue to be, important issues (van Dijk, Auger, Jaszcyszyn, & Thermes, 2014; O'Rawe, Ferson, & Lyon, 2015). Benchmarking and optimizing computational pipelines for experiments that use different markers and target aquatic taxa will be helpful for refining metabarcoding analytical guidelines. Testing with different markers may yield different recommendations in terms of sequence length—as it depends on marker length and variability of target regions—and quality filtration—as it depends on sequence length. Testing with different taxa may yield different results across the entire pipeline, depending on the marker used. Because of the prevalence of metabarcoding in current research (and accordingly, the prevalence of computational sequence processing), there is a need for more studies that deeply explore and optimize sequence processing pipelines for different applications. We advise users conducting biological invasions research with metabarcoding to test multiple parameter sets when processing data and, when possible, skip clustering or denoising. One can obtain a consensus from multiple runs with different parameters, improving confidence and gaining different perspectives of the data. In the context of early detection of AIS and across the range of parameters tested, we observed no situation where a parameter did not contribute to either false‐positive or false‐negative error in a significant manner (aside from singletons when denoising was used, Table S5). Thus, all parameters should be carefully considered in this context.

One important implication of our study is that, in metabarcoding, there will almost always be some false‐positive error and some false‐negative error. To fully eliminate false‐negative error—especially with low sequence abundance for some taxa, as is ideal in the context of early detection of AIS—there will almost surely be some false‐positive error and it can become a serious issue. Given the potential difficulty in balancing false‐positive and false‐negative errors in this context, does metabarcoding have a place in the early detection of AIS? We believe it does, although it may be difficult to confirm that a target AIS is in a sampled waterbody using metabarcoding (or a single marker) alone. A more effective strategy for conservation or AIS management applications would be to first use metabarcoding with the sequence processing strategy that we suggested, followed by a targeted genetic approach using highly species‐specific markers and primers (e.g., using COI) or traditional sampling methods to confirm the presence of the species with greater confidence. For a given combination of marker, target taxon, and sampling method, until a deep optimization is performed, analyzing sequence retention given length and filtering strength can provide some information with which to start a small search for good parameters.

## DATA ARCHIVING

Initial dataset, optimization code, simulation code and computed data are available on GitHub (https://github.com/ScottRyanD/AISDetection).

## CONFLICT OF INTEREST

None declared.
