# Supplementary material for: Optimization and performance testing of a sequence processing pipeline applied to detection of nonindigenous species
Source: Evol Appl. 2018 Feb 20;11(6):891–905. doi: 10.1111/eva.12604 (PMC5999198; doi:10.1111/eva.12604)

**Supplementary Material**

*Data S1: Description of the Bulk Sample Metabarcoding Process*

The process begins with a bulk sample, which often involves the use of specific nets to capture targets. Total genomic DNA is then extracted and amplified using primers that are specifically designed or selected for the study. The amplified DNA is then sequenced, and once the sequences are obtained this data can be subjected to computational processing that might involve processes like filtration, denoising, or clustering. Processed sequences are then run against reference databases to determine their taxonomic identity.

*Data S2: Mock Community Dataset (D1) Preparation*

D1 was a mock community of sequences developed using 20 AIS individuals, each of a different taxon that was individually tagged by adding short and unique sequences in the primers (Brown *et al.* 2015). This dataset was referred to as the “Tagged individual community” in the paper by Brown *et al.* (2015). The dataset originally contained 115902 sequences (unevenly distributed across the 20 taxa), with sequence length of approximately 400-600bp. This library was amplified using a primer pair developed by Zhan *et al.* (2013) and pyrosequenced using 454 GS-FLX Titanium platform (454 Life Sciences, Branford, CT, USA) by Genome Quebec (see Brown *et al.* 2015 for more details of library production). We removed all sequences of the invaders *Dreissena polymorpha* and *Ciona intestinalis* because preliminary analyses indicated that these samples were likely contaminated. We BLASTed all sequences of this dataset and found that roughly half of those from *D. polymorpha* and *C. intestinalis* aligned best with different taxa also found in this dataset. However, we also acquired 18S sequences of two other AIS: the green crab *Carcinus maenus*, which is a marine AIS of global importance, and the quagga mussel *Dreissena rostriformis bugensis*, which is a major problem in lakes in Europe and North America. We obtained green crab sequences from Brown *et al.* (2015), while those of quagga mussels were detected in bulk zooplankton samples (Chain *et al.* 2016). Both research groups used the same library production protocol and sequencing platform as described above, though the latter used a primer designed by Zhan, Bailey, *et al.* (2014). We refer to the dataset consisting of the sequences from the 18 taxa from the mock community, plus green crab and quagga mussel sequences, as D1. Therefore, D1 consisted of different abundances of sequences from 20 taxa with varied relatedness. This dataset was used for both the optimization and performance testing stages. For optimization and performance testing, we separated this dataset into 20 separate sequence sets, each consisting of sequences from a single taxon (Table S7). The amplified fragment for all 20 taxa was ≥ 400bp, and mean sequence length was 466bp.

*Data S3: Classification of Sequences*

When the pipeline was performed on a sample with a given parameter set, a set of OTUs was generated each of which had a representative sequence. These representative sequences were run against a reference database using an alignment search tool to determine their taxonomic identity. Basic Local Alignment Search Tool (BLAST - Altschul *et al.* 1990) is one such computational tool. We used the NCBI nucleotide database as our reference (retrieved June 2017) and BLASTn (BLAST for nucleotide sequences). We precomputed the class of each sequence so we could efficiently classify each OTU generated in optimization and performance testing based on its representative sequence. For optimization, this was necessary to evaluate the quality of each parameter set based on the OTUs it produced from the optimization samples. For performance testing, this was necessary to determine if an inoculated taxon could be correctly recovered from a sample.

We computed the list of all BLAST hits with ≥ 97% identity, which in BLASTn were sorted by decreasing hit similarity using the metrics E-value, bit-score, and identity. However, any number of hits may have had identical similarity scores using these metrics. Moreover, sequences (especially if they contained errors) may share more similarity with sequences of a different species than those of their own. Each of these situations made accurate identification of a sequence challenging. Worse yet, a sequence may not have aligned with sequences of its own species with sufficiently high alignment score, or the reference database may not have contained sequences of the queried species. Considering these complications, we classified each sequence in D1 as correct, incorrect, or ambiguous with the following definitions. A correct sequence was one that aligned best with a reference sequence of its true identity, with identity ≥ 97%, whether alignments to other taxa were tied in similarity score or not. An ambiguous sequence was one that aligned with a higher score to a reference sequence of a different taxon, though it still aligned to its correct taxon with identity ≥ 97%. An incorrect sequence aligned with a reference sequence of its true identity with identity < 97%.

To classify each sequence, we first had to establish a ground truth BLAST identity for each taxon using our reference database (Table S6). Of the 20 taxa, 11 BLAST identities matched their corresponding morphological identities to species and five matched to genus. Of the remainders, two taxa were assigned generic 18S metazoan identities, and two were assigned different identities altogether compared to their morphological identities. All samples in D1 obtained from Brown *et al.* (2016), where a sample is a set of sequences from a single taxon, were from specimens morphologically identified in that study. For these samples, if the majority of sequences aligned with reference sequences of their morphological identity with ≥ 97% identity, the morphological identity was assumed correct. Otherwise, the taxon with the highest BLAST similarity score was assumed correct. In most cases, BLAST identity of sequences matched their morphological identity, though in some cases they did not, mainly because the morphological identity did not exist in the reference database. Sequences of *Dreissena* and *Carcinus* were identified through BLAST in Chain *et al.* (2016).

For each taxon sample in D1, we generated five new samples by trimming the original sample to each of the lengths tested in this study (300bp, 325bp, 350bp, 375bp, and 400bp). We ran BLASTn on each of the trimmed samples for each taxon with a 97% identity cutoff. We then parsed all the BLAST results and classified each sequence according to the definitions above. To save these classifications, we generated three “BLAST cache” files for each taxon-length combination – one for correct, one for ambiguous, and one for incorrect sequences. In these files, we wrote the sequence labels for all sequences of the given class, for fast reference in the future. With a given OTU from optimization or performance testing, we could then search the cache files for the matching sequence label to determine its class.

*Data S4: Parameters and Values Used in Previous Studies*

Generally, we tested more lenient parameter values than those used in related studies (Bokulich *et al.* 2013; Pawlowski *et al.* 2014; Elbrecht and Leese 2015; Flynn *et al.* 2015; Brown *et al.* 2015; Brown *et al.* 2016; Chain *et al.* 2016; Hänfling *et al.* 2016; Port *et al.* 2016; Bista *et al.* 2017) because our reads were comparatively long and variable in quality, and sequence quality decreases with sequence length. Aside from the studies by Brown *et al.* (2015) and Elbrecht and Leese (2015), the minimum length cutoff used in all related studies was less than 300bp. Q filters ranged from 20-30 depending on strategy (per base call, sliding window, mean across full sequence *etc.*) and sequence length. MEE filters ranged from 0.5-1.0 (Flynn *et al.* 2015; Brown *et al.* 2015; Port *et al.* 2016; Bista *et al.* 2017). Clustering identity thresholds ranged from 97%-99% across a variety of clustering algorithms (Flynn *et al.* 2015; Brown *et al.* 2015; Brown *et al.* 2016; Chain *et al.* 2016; Port *et al.* 2016; Bista *et al.* 2017; Clarke *et al.* 2017). Some studies discarding singletons while others kept them (Elbrecht and Leese 2015; Flynn *et al.* 2015; Brown *et al.* 2015; Brown *et al.* 2016; Chain *et al.* 2016; Port *et al.* 2016; Bista *et al.* 2017; Clarke *et al.* 2017). The UNOISE3 denoising algorithm (Edgar 2016) was more recently developed and thus none of the aforementioned studies used this algorithm.

*Data S5: Parameter Set Ranking*

In optimization, we computed the number of OTUs of a fourth class (in addition to correct, ambiguous, and incorrect) – redundant – which meant that we already had a correct or ambiguous OTU for a particular taxon. For a given taxon, if we had a correct and an ambiguous OTU, the correct OTU took precedence and the ambiguous OTU was reclassified to redundant. Thus, a sample could yield at most 20 correct or ambiguous OTUs in total (one for each taxon), and any remaining correct or ambiguous OTUs were considered redundant. We considered the number of redundant OTUs in optimization for two reasons. First, it could take significantly more processing (manual work) to determine the identity of an OTU, particularly if it was correct but had multiple high-scoring hits in BLAST, or if it was ambiguous. Secondly, more computational time would have been necessary for downstream analysis if there were more OTUs with which to work. In each part of the optimization stage, to find optimal sets, we ranked the parameter sets in order of decreasing optimality based on the following criteria.

In both parts of the optimization process, parameter set *a* was considered more optimal than parameter set *b* if the former’s total number of correct and ambiguous OTUs was greater than the latter’s. In the case of a tie, the parameter set with the greater number of correct OTUs was more optimal. Missing correct or ambiguous OTUs constitutes a false negative error, which is problematic in estimating species richness but potentially catastrophic in early detection of AIS. If two parameter sets were still tied, the parameter set with the fewest incorrect OTUs was considered more optimal. The more incorrect OTUs generated by a parameter set, the more likely a user could have been to commit a false positive error using that parameter set. If the number of incorrect OTUs was equal as well, the parameter set with fewer redundant OTUs was more optimal. If all OTU counts were equal, the parameter sets performed equally. For each part of the optimization process, we grouped the optimization results by parameter sets that performed clustering, denoising, or neither so that we could compare these three sequence processing methods.

*Data S6: Optimization Results*

We analyzed the classification of sequences in the samples we used for optimization to determine whether all taxa could be recovered and to determine how many incorrect OTUs would be generated (false positive errors). Classification of sequences varied with length, so we analyzed all optimization samples trimmed to each of the lengths tested. The single sample we used to optimize species richness estimation (dataset D1) ranged from 749 incorrect sequences at length 400bp to 1484 at length 325bp, with the remaining sequences classified as correct or ambiguous. At most, only 19 taxa could possibly be recovered (18 correct and one ambiguous) using sequence lengths of 300bp and 325bp, whereas at lengths 350bp, 375bp, and 400bp all 20 taxa could be recovered (19 correct and one ambiguous). The samples we used to optimize for early detection of AIS ranged from a mean of 53.9 (SD = 2.1) incorrect sequences at length 325bp to 25.9 (SD = 4.0) at length 400bp, with the remaining sequences classified as correct or ambiguous. The mean total number of taxa that could be recovered was 19.00 (SD = 0.00) at lengths 300bp and 325bp (18 correct and one ambiguous), 19.97 (SD = 0.17) at length 350bp (18.97 correct and one ambiguous) and 20 (SD = 0) at lengths 375bp, and 400bp.

When optimizing for species richness estimation without clustering or denoising, incorrect OTUs ranged from 19 to 613 for 27 parameter sets that recovered all taxa (Figure S2a). With clustering, 18 parameter sets that recovered all taxa yielded from four to 184 incorrect OTUs (Figure S2b). Only the top 22 parameter sets using denoising recovered all 20 taxa without any incorrect OTUs (Figure S2c). With denoising, 46 parameter sets recovered all taxa with a maximum of 8 incorrect OTUs. When optimizing for early detection of AIS, no parameter set recovered all taxa without allowing some incorrect OTUs to pass through. Without clustering or denoising, 13 parameter sets recovered all taxa however they yielded a mean of 11.1 incorrect OTUs as well (Figure S3a). Further, 43 parameter sets without clustering or denoising recovered at least 19 taxa on average. No parameter set involving clustering recovered all taxa in all replicates; however, 15 recovered a mean of over 19 taxa and yielded a mean of 3.5 incorrect OTUs with a maximum of 9.1 incorrect OTUs (Figure S3b). With denoising, the top four parameter sets recovered all taxa while only yielding at most a mean of 0.25 incorrect OTUs (Figure S3c).

*Data S7: Discussion of Clustering and Denoising*

Typically, 97% is considered a standard for clustering identity thresholds (see Edgar 2013). Through optimization, we found that 99% clustering identity performed better for zooplankton using the 18S V4 fragment. There were two highly related taxa, *Carcinus* and *Cancer,* which impacted the optimality of parameter sets using clustering. In practice, this situation may occur where two distinct species in a sample share very high identity (> 97%). Users of such sequence processing pipelines will not know in advance what the appropriate clustering threshold is, as it depends on the relatedness of taxa in their sample, and incorrect assignment of the threshold can be a source of errors that have dire consequences (particularly for early detection of AIS; Brown *et al.* 2015). In our study, sensitivity and detectability were reduced when clustering because clusters form that contain sequences from both the community sample and AIS sequences that we introduced to the sample. In real applications of this pipeline, clustering may create clusters with sequences from more than one species, hiding sequences of taxa and inadvertently rendering them undetectable downstream. Fortunately, with modern computers, clustering when a reference database exists is often unnecessary; one could use a parallel computing strategy (*e.g.* in BLAST) that could reduce computational time and keep processing of metabarcoding data tractable. Our findings suggest that clustering reduces taxonomic resolution and removes potentially informational sequences from the dataset prior to taxonomic assignment. Thus, we support suggestions of Brown *et al.* (2016) and Chain *et al.* (2016) to avoid clustering altogether if early detection of AIS is the project goal. However, false positive errors were reduced by clustering spurious reads with their correct counterparts, which is especially beneficial when estimating species richness. Thus, if clustering is necessary or desired, we recommend using a higher similarity threshold than what has been classically used – 99% instead of 97% – to reduce false negatives while simultaneously reducing spurious OTUs.

With respect to denoising, the current version of USEARCH uses UNOISE3, which is a relatively new algorithm and is technically a form of clustering itself. Its likeness to clustering was evident in its performance. In terms of detectability, the combinations of taxa and ports that clustering struggled with were nearly the same as denoising, though the latter performed slightly worse. For problematic combinations of taxa and ports, denoising usually fared worse than clustering. Further, denoising yielded slightly lower sensitivity than clustering. For early detection of AIS, these characteristics are potentially problematic. False negative errors could occur by incorrectly flagging valid sequences as noise. On the other hand, with respect to species richness estimates, incorrect sequences were removed more effectively through denoising than through any other processing method. The default minimum abundance threshold was 8, which we found worked very well for conducting species richness estimates. However, we also found that this default threshold was not viable when aiming for early detection of AIS. Thus, our recommendation for denoising is like that of clustering. If this pipeline is being used for early detection of AIS, either avoid denoising or use a conservative minimum abundance threshold (for instance, minimum abundance of 2-4 rather than 8). If, on the other hand, the pipeline is being used to estimate species richness, denoising with a minimum abundance threshold of eight will remove a high proportion of spurious reads and serve to reduce false positives. The denoising algorithm of USEARCH does allow users to save all OTUs (including those flagged as chimeric or noisy). Thus, an alternative is to denoise but be cognizant that some sequences may be wrongly flagged as chimeric or noisy. Then, further analysis could then reduce false negatives even after denoising (*e.g.* by running BLAST with the chimeric or noisy sequences, aligning them against denoised OTUs, or applying an evolutionary model to the denoised OTUs and chimeric or noisy sequences).

Table S1: Dataset D2, containing sequences of ten Canadian ports sampled (see Chain *et al.* 2016). Number of sequences and proportion of sequences kept at length 350 bp given a Phred score (Q) filter of 10 and 20 or MEE filter of 1 are shown. Samples ranged greatly in quality and abundance. Churchill and Halifax yielded sequences of relatively low quality, whereas Hawkesbury, Sept Iles, and Thunder Bay yielded sequences of relatively high quality. With a Phred score filter of 20, no sequences of Churchill or Halifax are retained.

| **Location** | **Sequences** | **Q = 10** | **Q = 20** | **MEE = 1** |
| --- | --- | --- | --- | --- |
| Churchill | 684163 | 0.2290 | 0.0000 | 0.0809 |
| Halifax | 877078 | 0.2480 | 0.0000 | 0.0477 |
| Hamilton | 686064 | 0.2660 | 0.0230 | 0.1750 |
| Hawkesbury | 444315 | 0.6370 | 0.1110 | 0.5076 |
| Nanaimo | 406215 | 0.6240 | 0.0200 | 0.4074 |
| Nanticoke | 480962 | 0.5820 | 0.0570 | 0.4305 |
| Sept Iles | 249663 | 0.9550 | 0.1900 | 0.8645 |
| Thunder Bay | 556984 | 0.6910 | 0.1170 | 0.5798 |
| Vancouver | 1008358 | 0.2670 | 0.0020 | 0.1359 |
| Victoria | 456391 | 0.5720 | 0.0310 | 0.3976 |

Table S2: Synopsis and values used of the six sequence processing parameters tested in this study. In total, 1050 parameter sets were tested in the optimization stage. Clustering and denoising steps were optional and mutually exclusive.

| **Parameter** | **Synopsis** | **Values Tested** |
| --- | --- | --- |
| Sequence Length | Length of sequences – shorter sequences discarded, longer sequences trimmed | 300, 325, 350, 375, 400 |
| Minimum Phred Score (Q) | Minimum quality score per base call | 10, 20, 30 |
| Maximum Expected Error (MEE) | Sequence-wide expected error score | 1.0, 1.5, 2.0, 2.5, 3.0 |
| Clustering Identity Threshold (Optional) | Intraspecific genetic identity threshold | 97%, 98%, 99% |
| Denoising Minimum Abundance Threshold (Optional) | Minimum abundance of a sequence to not be considered noise | 2, 4, 8 |
| Singletons | Do we keep unique sequences | Yes, no |

Table S3: Selected high-ranking parameter sets from optimization using all sequences in D1 in a single sample. This optimization aimed to determine parameter sets that most accurately reconstruct a community with low false positive error. We selected four parameter sets per processing method (clustering, denoising, or neither) by selecting the best one and subsequently selecting those that had at least two parameters different from any previously selected (to reduce redundancy for performance testing). Correct operational taxonomic units (OTUs) were those that BLASTed to the assumed identity with rank 1 (using the BLASTn default sort method) and identity > 97%. Ambiguous OTUs were those that BLASTed to the assumed identity with rank > 1 and identity > 97%. Incorrect OTUs were those that did not BLAST to the assumed identity with identity > 97%. Correct and ambiguous OTUs were combined here because the correct identity of ambiguous OTUs could be determined downstream. Trim length is the length of sequences in base pairs (bp), Q filter is the strength of the Phred score filter, MEE filter is the strength of the maximum expected errors filter, and clustering ID is the clustering identity threshold. For processing methods, C represents Clustering, D represents Denoising, and NCOD represents No Clustering Or Denoising. These parameter sets were used for performance testing. Overall, 17/1050 parameter sets recovered all 20 AIS with only 50 sequences from each taxon in all 100 replicates.

| **Optimized for Accurate Species Richness Estimates** | | | | | | | | |
| --- | --- | --- | --- | --- | --- | --- | --- | --- |
| **Trim Length (bp)** | **Q Filter** | **MEE Filter** | **Processing Method** | **Clustering ID (%) or Denoising Minimum Abundance** | **Singletons** | **Correct + Ambiguous OTUs** | **Incorrect OTUs** | **Redundant OTUs** |
| 375 | 10 | 1.5 | D | 8 | No | 20 | 0 | 15 |
| 375 | 10 | 3.0 | D | 8 | Yes | 20 | 0 | 16 |
| 400 | 10 | 2.0 | D | 8 | No | 20 | 0 | 24 |
| 400 | 10 | 2.5 | D | 8 | Yes | 20 | 0 | 25 |
| 400 | 10 | 1.5 | C | 99 | No | 20 | 4 | 59 |
| 375 | 10 | 2.0 | C | 99 | No | 20 | 8 | 110 |
| 400 | 10 | 1.5 | NCOD | N/A | No | 20 | 19 | 4250 |
| 400 | 10 | 1.0 | C | 99 | Yes | 20 | 32 | 562 |
| 375 | 10 | 2.0 | NCOD | N/A | No | 20 | 36 | 4345 |
| 350 | 10 | 2.5 | NCOD | N/A | No | 20 | 54 | 4299 |
| 375 | 10 | 1.5 | C | 99 | Yes | 20 | 78 | 1826 |
| 400 | 10 | 1.0 | NCOD | N/A | Yes | 20 | 95 | 14155 |

Table S4: Selected high-ranking parameter sets from optimization with 50 sequences of each taxon from D1 per sample, with 100 replicates. This optimization aimed to determine parameter sets that yielded high sensitivity. We selected four parameter sets per processing method (clustering, denoising, or neither) by selecting the best one and subsequently selecting those that had at least two parameters different from any previously selected (to reduce redundancy for performance testing). Correct operational taxonomic units (OTUs) were those that BLASTed to the assumed identity with rank 1 (using the BLASTn default sort method) and identity > 97%. Ambiguous OTUs were those that BLASTed to the assumed identity with rank > 1 and identity > 97%. Incorrect OTUs were those that did not BLAST to the assumed identity with identity > 97%. Correct and ambiguous OTUs were combined here because the correct identity of ambiguous OTUs could be determined downstream. Trim length is the length of sequences in base pairs (bp), Q filter is the strength of the Phred score filter, MEE filter is the strength of the maximum expected errors filter, and clustering ID is the clustering identity threshold. For processing methods, C represents Clustering, D represents Denoising, and NCOD represents No Clustering Or Denoising. These parameter sets were used for performance testing. Overall, 17/1050 parameter sets recovered all 20 AIS with only 50 sequences from each taxon in all 100 replicates.

| **Optimized for Early Detection of AIS** | | | | | | | | |
| --- | --- | --- | --- | --- | --- | --- | --- | --- |
| **Trim Length (bp)** | **Q Filter** | **MEE Filter** | **Processing Method** | **Clustering ID (%) or Denoising Minimum Abundance** | **Singletons** | **Correct + Ambiguous OTUs** | **Incorrect OTUs** | **Redundant OTUs** |
| 400 | 10 | 3 | D | 2 | No | 20 | 0.2 | 21.3 |
| 400 | 10 | 2.5 | D | 2 | Yes | 20 | 0.3 | 21.1 |
| 400 | 10 | 2.5 | NCOD | N/A | No | 20 | 1.0 | 59.2 |
| 375 | 10 | 2 | NCOD | N/A | No | 20 | 2.3 | 50.6 |
| 400 | 10 | 1.5 | NCOD | N/A | Yes | 20 | 8.2 | 349.1 |
| 375 | 10 | 2.5 | NCOD | N/A | Yes | 20 | 22.6 | 409.1 |
| 400 | 10 | 2.5 | C | 99 | Yes | 19.8 | 5.8 | 37.0 |
| 375 | 10 | 3 | D | 2 | Yes | 19.75 | 0.4 | 18.8 |
| 375 | 10 | 2.5 | D | 2 | No | 19.73 | 0.4 | 18.7 |
| 375 | 10 | 3 | C | 99 | Yes | 19.6 | 9.1 | 61.9 |
| 400 | 10 | 3 | C | 99 | No | 19.3 | 0.0 | 1.4 |
| 375 | 10 | 2.5 | C | 99 | No | 19.0 | 0.3 | 2.6 |

Table S5: Coefficients and *p* values for multiple regression given standardized parameter values to predict the number of correct + ambiguous OTUs and the number of incorrect OTUs for each sequence processing method and for each research goal. Coefficient magnitude signifies importance of the corresponding parameter in determining the predicted value, and *p* value indicates significance of impact. “Q” denotes Q filter, “Length” denotes sequence length, “Singletons” denotes whether singletons were kept or discarded, “MEE” denotes maximum expected error filter, “ID” denotes clustering identity threshold, and “DMA” denotes denoising minimum abundance.

|  |  |  | Correct + Ambiguous | | Incorrect | |
| --- | --- | --- | --- | --- | --- | --- |
| Research Goal | Processing Method (adj. r-squared correct + ambiguous, adj. r-squared incorrect) | Parameter | Coefficient | p value | Coefficient | p value |
| Species Richness | Clustering (0.80, 0.41) | Length | -0.474 | <0.001 | -4.29 | 0.007 |
|  |  | Q | -5.746 | <0.001 | -21.21 | <0.001 |
|  |  | MEE | 0.035 | 0.797 | 6.47 | <0.001 |
|  |  | ID | 0.163 | 0.225 | 6.46 | <0.001 |
|  |  | Singletons | -0.42 | 0.002 | -15.46 | <0.001 |
|  | Denoising  (0.89, 0.51) | Length | -0.5 | <0.001 | -0.494 | <0.001 |
|  |  | Q | -6.294 | <0.001 | -1.5749 | <0.001 |
|  |  | MEE | 0.091 | 0.399 | 0.1542 | 0.082 |
|  |  | DMA | -0.995 | <0.001 | -0.9596 | <0.001 |
|  |  | Singletons | <0.001 | 1 | <0.001 | 1 |
|  | Neither (0.80, 0.47) | Length | -0.44 | 0.085 | -12.97 | 0.165 |
|  |  | Q | -6.12 | <0.001 | -86.02 | <0.001 |
|  |  | MEE | 0.028 | 0.911 | 26.11 | 0.006 |
|  |  | Singletons | -0.421 | 0.099 | -59.2 | <0.001 |
| Early Detection of AIS | Clustering (0.95, 0.48) | Length | -0.5478 | <0.001 | -0.2049 | 0.001 |
|  |  | Q | -7.1928 | <0.001 | -1.0415 | <0.001 |
|  |  | MEE | 0.065 | 0.404 | 0.2399 | <0.001 |
|  |  | ID | 0.107 | 0.169 | 0.2183 | 0.001 |
|  |  | Singletons | -0.8472 | 0 | -0.664 | 0 |
|  | Denoising (0.94, 0.55) | Length | -0.6302 | 0 | -0.1134 | 0 |
|  |  | Q | -7.3123 | 0 | -0.2579 | 0 |
|  |  | MEE | 0.1327 | 0.13 | 0.024 | 0.049 |
|  |  | DMA | -1.4029 | 0 | -0.0425 | 0.001 |
|  |  | Singletons | 0 | 1 | 0 | 1 |
|  | Neither (0.95, 0.50) | Length | -0.52 | 0.001 | -0.762 | 0.077 |
|  |  | Q | -7.627 | 0 | -4.331 | 0 |
|  |  | MEE | 0.065 | 0.661 | 1.149 | 0.008 |
|  |  | Singletons | -0.873 | 0 | -2.643 | 0 |

Table S6: Morphological and assumed BLAST identities of sequences from dataset D1, separated by taxon. Correct sequences were those that BLASTed to the assumed identity with rank 1 (using the BLASTn default sort method) and identity > 97%. Ambiguous sequences were those that BLASTed to the assumed identity with rank > 1 and identity > 97%. Incorrect sequences were those that did not BLAST to the assumed identity with identity > 97%. All taxa except *Epischura*, *Mesocyclops*, and *Senecella* had fewer than 1% incorrect sequences. For *Mesocyclops*, most sequences that were labelled incorrect did BLAST to *Mesocyclops*, but with an identity of less than 97%. *Carcinus* sequences exhibited high identity with many reference sequences.

| **Morphological Identity** | **Assumed BLAST Identity** | **Correct Sequences** | **Ambiguous Sequences** | **Incorrect Sequences** | **Correct + Ambiguous (%)** | **Incorrect (%)** |
| --- | --- | --- | --- | --- | --- | --- |
| *Artemia salina* | *Artemia salina* | 2137 | 0 | 8 | 99.6 | 0.4 |
| *Balanus crenatus* | *Balanus crenatus* | 14724 | 0 | 8 | 99.9 | 0.1 |
| *Brachionus calyciflorus* | *Brachionus calyciflorus* | 207 | 0 | 0 | 100.0 | 0.0 |
| *Cancer sp.* | *Cancer sp.* | 1620 | 4 | 5 | 99.7 | 0.3 |
| *Carcinus maenas* | *Carcinus maenas* | 43 | 157 | 0 | 100.0 | 0.0 |
| *Cercopagis pengoi* | *Cercopagis pengoi* | 1217 | 0 | 5 | 99.6 | 0.4 |
| *Corbicula fluminea* | *Corbicula fluminea* | 46842 | 0 | 73 | 99.8 | 0.2 |
| *Daphnia mendotae* | *Daphnia sp.* | 694 | 11 | 1 | 99.9 | 0.1 |
| *Diacyclops thomasi* | *Diacyclops bicuspidatus* | 0 | 812 | 0 | 100.0 | 0.0 |
| *Dreissena rostriformis bugensis* | *Dreissena rostriformis bugensis* | 200 | 0 | 0 | 100.0 | 0.0 |
| *Echinogammarus ischnus* | *Chaetogammarus ischnus* | 7280 | 0 | 57 | 99.2 | 0.8 |
| *Epischura lacustris* | *Eurytemora affinis* | 9628 | 248 | 126 | 98.7 | 1.3 |
| *Leptodiaptomus ashlandi* | *Leptodiaptomus ashlandi* | 5414 | 25 | 21 | 99.6 | 0.4 |
| *Mesocyclops edax* | *Mesocyclops pehpeiensis* | 56 | 0 | 999 | 5.3 | 94.7 |
| *Microsetella norvegica* | *Uncultured Metazoan Partial* | 809 | 2 | 3 | 99.6 | 0.4 |
| *Oikopleura labradoriensis* | *Uncultured Eukaryote 18S* | 3543 | 0 | 2 | 99.9 | 0.1 |
| *Palaemonetes sp.* | *Palaemonetes sp.* | 5163 | 1 | 6 | 99.9 | 0.1 |
| *Pleuroxus denticulatus* | *Pleuroxus denticulatus* | 642 | 0 | 2 | 99.7 | 0.3 |
| *Senecella calanoides* | *Euchirella sp.* | 340 | 2 | 6 | 98.3 | 1.7 |
| *Themisto libellula* | *Themisto libellula* | 4246 | 1 | 11 | 99.5 | 0.3 |

Table S7: Dataset D1, with sequences grouped by taxon. Proportion of sequences kept at length 350 bp given a Phred score (Q) filter or MEE filter of varying strengths are shown as a proxy of dataset quality. Sequences ranged greatly in quality and abundance, with *Brachionus* and *Mesocyclops* yielding sequences of lowest quality. With a Phred score filter of 20, no sequences of *Brachionus* or *Mesocyclops* were retained.

| **Taxon** | **Sequences** | **Q = 10** | **Q = 20** | **MEE = 1** |
| --- | --- | --- | --- | --- |
| *Artemia salina* | 2145 | 0.9920 | 0.0490 | 0.8015 |
| *Balanus crenatus* | 14732 | 0.9910 | 0.1310 | 0.8128 |
| *Brachionus calyciflorus* | 207 | 0.9950 | 0.0000 | 0.0483 |
| *Cancer sp.* | 1629 | 0.9940 | 0.1040 | 0.7185 |
| *Carcinus maenas* | 200 | 1.0000 | 0.1750 | 0.9400 |
| *Cercopagis pengoi* | 1222 | 0.9920 | 0.0110 | 0.7709 |
| *Corbicula fluminea* | 46915 | 0.9900 | 0.2980 | 0.8952 |
| *Daphnia mendotae* | 706 | 0.9750 | 0.0160 | 0.6232 |
| *Diacyclops thomasi* | 812 | 0.9900 | 0.0090 | 0.7106 |
| *Dreissena rostriformis bugensis* | 200 | 1.0000 | 0.1550 | 0.9450 |
| *Echinogammarus ischnus* | 7337 | 0.9820 | 0.2430 | 0.8327 |
| *Epischura lacustris* | 10002 | 0.9900 | 0.1400 | 0.8465 |
| *Leptodiaptomus ashlandi* | 5461 | 0.9890 | 0.0790 | 0.7539 |
| *Mesocyclops edax* | 1055 | 0.9910 | 0.0000 | 0.2812 |
| *Microsetella norvegica* | 814 | 0.9950 | 0.0530 | 0.8136 |
| *Oikopleura labradoriensis* | 3545 | 0.9940 | 0.1090 | 0.8434 |
| *Palaemonetes sp.* | 5170 | 0.9930 | 0.3630 | 0.9154 |
| *Pleuroxus denticulatus* | 644 | 0.9800 | 0.0080 | 0.6182 |
| *Senecella calanoides* | 348 | 0.9970 | 0.0140 | 0.4580 |
| *Themisto libellula* | 4269 | 0.9830 | 0.5000 | 0.9311 |

Figure S1: Distributions of number of sequences required (x-axis) to detect target taxa in community samples for parameter sets optimized for the early detection of AIS using no clustering or denoising. Frequency was the number of simulations in which the target was detected at a given number of sequences inoculated. Each color represents a selected parameter set. Parameter sets are in the format “length/Q filter/MEE filter/minimum abundance”.

Figure S2: Number of correct + ambiguous and incorrect OTUs for parameter sets optimized for accurate estimates of species richness using no clustering or denoising (a), clustering (b), and denoising (c), by optimization rank. Note the difference in scale on the y axis and that we tested fewer parameter sets using no clustering or denoising.

Figure S3: Number of correct + ambiguous and incorrect OTUs for parameter sets optimized for high sensitivity using no clustering or denoising (a), clustering (b), and denoising (c), by optimization rank. Note that we tested fewer parameter sets using no clustering or denoising.

Figure S1


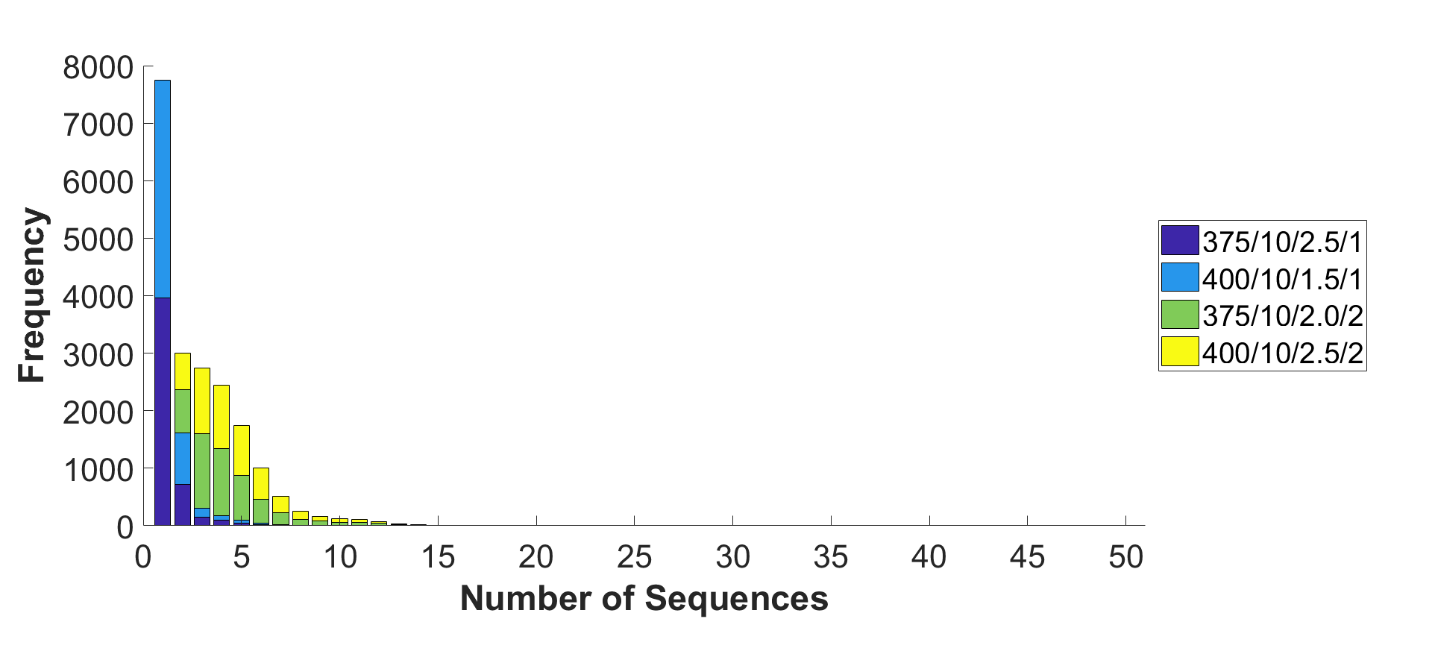


Figure S2


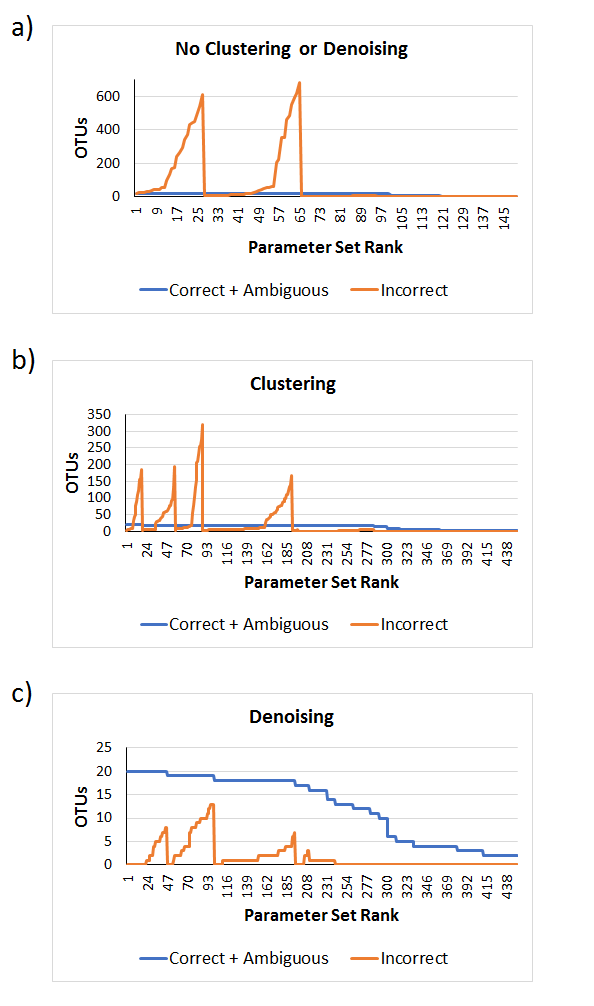


Figure S3


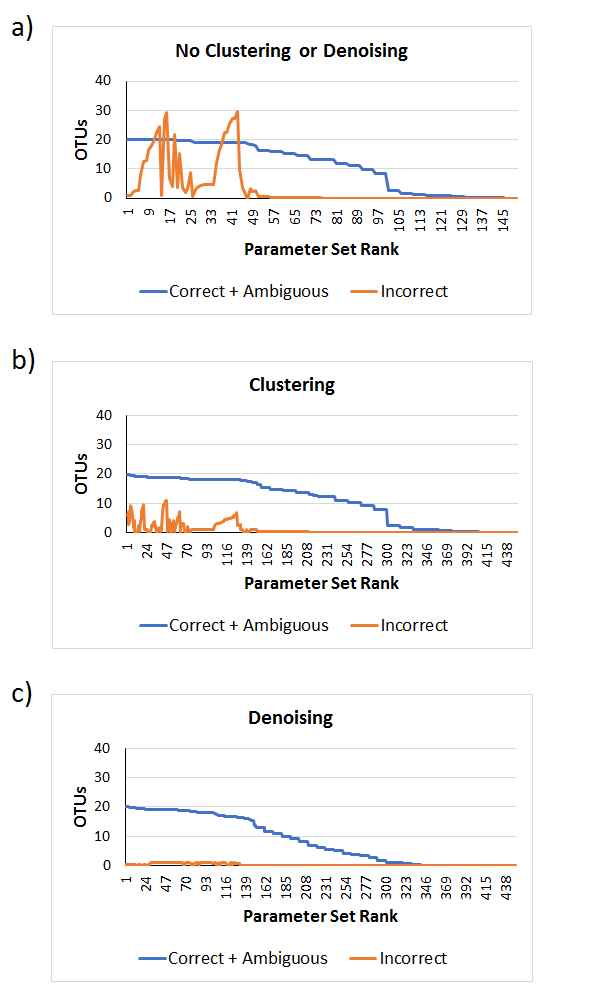

Supplement: Supplementary file 1 [file EVA-11-891-s001.docx]
